# A Deep Learning-Enhanced Compartmental Model and Its Application in Modeling Omicron in China

**DOI:** 10.3390/bioengineering11090906

**Published:** 2024-09-10

**Authors:** Qi Deng, Guifang Wang

**Affiliations:** 1 College of Artificial Intelligence, Hubei University of Automotive Technology, Shiyan 442002, China; 2 Jack Welch College of Business and Technology, Sacred Heart University, Fairfield, CT 06825, USA; 3 Department of Respiratory Diseases and Critical Medicine, Huashan Hospital, Fudan University, Shanghai 200040, China; wangguifang@fudan.edu.cn; 4 Department of Respiratory Diseases and Critical Medicine, Quzhou Hospital, Wenzhou Medical University, Quzhou 325015, China

**Keywords:** epidemics, COVID-19, Omicron, compartmental model, transmission parameter, deep learning

## Abstract

The mainstream compartmental models require stochastic parameterization to estimate the transmission parameters between compartments, whose calculation depend upon detailed statistics on epidemiological characteristics, which are expensive, economically and resource-wise, to collect. In addition, infectious diseases spread in three dimensions: temporal, spatial, and mobile, i.e., they affect a population through not only the time progression of infection, but also the geographic distribution and physical mobility of the population. However, the parameterization process for the mainstream compartmental models does not effectively capture the spatial and mobile dimensions. As an alternative, deep learning techniques are utilized in estimating these stochastic parameters with greatly reduced dependency on data particularity and with a built-in temporal–spatial–mobile process that models the geographic distribution and physical mobility of the population. In particular, we apply DNN (Deep Neural Network) and LSTM (Long-Short Term Memory) techniques to estimate the transmission parameters in a customized compartmental model, then feed the estimated transmission parameters to the compartmental model to predict the development of the Omicron epidemic in China over the 28 days for the period between 4 June and 1 July 2022. The average levels of predication accuracy of the model are 98% and 92% for the number of infections and deaths, respectively. We establish that deep learning techniques provide an alternative to the prevalent compartmental modes and demonstrate the efficacy and potential of applying deep learning methodologies in predicting the dynamics of infectious diseases.

## 1. Introduction

The Omicron variant of COVID-19 started invading China as early as November 2021 and became a full-fledged epidemic in late February 2022. If one assumes that all confirmed infections since 31 November 2022 have been Omicron cases, as of 3 June 2022, there have been 764,369 confirmed Omicron cases and 590 patients have died from Omicron infection in mainland China. The numbers of confirmed and deceased cases are 1,214,192 and 9382, respectively, in Hong Kong. For Taiwan, the numbers are 2,274,666 and 2663.

Most of the research on Omicron focuses on the effectiveness of immunization, vaccination, and treatment, with relatively few epidemiological studies on the variant, especially in a Chinese context. A computational simulation–model study that utilized a customized Monte Carlo model to estimate the effect of facemask use before and after different COVID-19 vaccination coverage levels was conducted [1]. A set of posterior statistical models to estimate cumulative infections and the cumulative proportion of the population in various locations has been produced [2]. While epidemiological studies on Omicron are lacking in general, there have been attempts to model the original variant dynamics in China. A classic SEIR model was used to infer the basic reproduction ratio and to simulate the Wuhan epidemic [3]. More sophisticated models have been developed to correlate the risk levels of foreign countries based on their travel exposure to China [4,5], including a stochastic dual-SEIR approach on both the Wuhan population and international travelers to estimate how transmission has varied over time from Wuhan to international destinations [5]. Simulations on international spread after the start of the travel ban from Wuhan on 23 January 2020 have also been conducted [6], which apply the Global Epidemic and Mobility Model (GLEAM) to a multitude of Chinese and global cities and a SEIR variety (SLIR) to project the impact of human-to-human transmissions.

Since March 2020, with the outbreak of the original variant declining in China, researchers have dedicated more efforts to analyzing the effectiveness of containment measures. Mobility and travel history data from Wuhan have been used to ascertain the impact of the drastic control measures implemented in China [7]. The spread and control of COVID-19 among Chinese cities with data on human movements and public health interventions has been investigated [8]. A transmission model to study the impact of social distancing and school closure has been built, utilizing the contact data for Wuhan and Shanghai and tracing information from Hunan [9].

In late February 2022, China, especially Shanghai, was hit hard by the Omicron variant. With a much higher initial reproduction rate than those of the original and Delta variants, at a value between 1.72 and 8.2 [10,11,12], the Omicron variant presented a potentially game-changing challenge to the country’s zero-COVID strategy that was effective against the original and Delta variants. An age-structured stochastic compartmental model (SLIRS) calibrated on the initial growth phase for the 2022 Omicron outbreak in Shanghai was developed [13]. The key contribution of the model is the inclusion of age-specific vaccine coverage data, vaccine efficacy against different clinical endpoints, the waning of immunity, different antiviral therapies, and nonpharmaceutical interventions.

The aim of this paper is to establish an innovative compartmental model, in which the transmission parameters are estimated by a family of multivariate, multistep deep learning methodologies. In particular, we apply DNN (deep neural network) and LSTM (long short-term memory) techniques to estimate the transmission parameters of a customized compartmental model and then feed the estimated transmission parameters to the compartmental model to predict and simulate the development of the Omicron epidemics in China (both including and excluding Hong Kong and Taiwan), as well as Hong Kong and Taiwan, for a 28-day period between 3 June and 1 July 2022. We use two datasets in our study: (1) a JHU CSSE dataset that tracks confirmed cases and deceased cases between 22 January 2020 and 7 June 2022 (863 days) to form training data for deep learning; and (2) a Tencent dataset that records daily cases (confirmed, active, deceased, recovered, etc.) to calculate and construct the transmission parameters for the compartmental model. We then compare the reported numbers of infections and deaths to measure the prediction accuracy of our models. The average levels of prediction accuracy provided by the models are 98% and 92% for the numbers of infections and deaths, respectively.

Our model provides an effective alternative to the prevalent compartmental models that depend upon detailed statistics on epidemiological transmission characteristics to establish the transmission parameters, yet are rather limited in modeling the geographic distribution and mobility of the population, which is the case for COVID-19 (see Section 2—Literature Review). The DNN and LSTM deep learning techniques that we apply are effective in estimating these stochastic parameters with greatly reduced dependency on data particularity and introduce a built-in temporal–spatial–mobile parameterization process that models the geographic distribution and mobility of the population. Our model demonstrates the efficacy and potential of applying deep learning methodologies in predicting the dynamics of infectious diseases.

## 2. Literature Review

### 2.1. Review of Compartmental Models

Modern epidemic modeling can be traced back to the Kermack–McKendrick (Kermack and McKendrick, 1927, 1932, 1933) theories [14,15,16]. Virtually all later compartmental models seek to improve upon or refine these earlier works, especially the original Kermack–McKendrick (1927) model [14]. A simplified version (which assumes that the transmission rate and recovery rate are constants) of the Kermack–McKendrick (1927) model [14] is also the direct predecessor of the most typical compartmental model, the SIR (Susceptible–Infectious–Recovered) model.

Currently, the prevailing compartmental model, the SEIR (Susceptible–Exposed–Infectious–Recovered) model (e.g., [17,18]), explicitly factors in that many infectious diseases have significant incubation periods. An individual is in the exposed compartment if they have had contact(s) with an infectious person (or infectious persons) but have not yet progressed to the infectious stage.

### 2.2. Review of Coronavirus Modeling

The COVID-19 virus that has ravaged China (and elsewhere) since December 2019 is of the coronavirus type. The coronavirus family also includes the infamous SARS-CoV and MERS-CoV infections that are responsible for the other most recent respiratory illnesses, namely, the SARS outbreak of 2003 and the MERS outbreak of 2013, respectively.

Acknowledging that SARS epidemics are geographically localized, Riley et al. [19] studied the SARS spread in Hong Kong by using a SEIR variety that accounts for the uneven spatial distribution of the infection. Huang et al. [20] proposed a small-world model that makes use of daily-contact social networks to simulate the dynamics of SARS transmission in Singapore, Taipei, and Toronto, with direct SIR and SEIR approaches. Small and Tse [21] proposed a SEIR with a significant incubation period in a small-world framework to simulate the localized SARS outbreak in Hong Kong. Masuda et al. [22] also formulated their model to account for super-spreaders in a small-world context. Small et al. [23] utilized SEIR and described a stochastic small-world network of localized and long-range links to analyze SARS outbreaks and the impact of superspreaders in Hong Kong.

Chowell et al. [24] established a SEIR variation, the SEIJR (susceptible, exposed, infective, diagnosed, recovered) model to analyze SARS outbreaks in Toronto, Hong Kong, and Singapore. Naheed et al. [25] used a numerical approach based on the SEIJR model that distinguishes infected and diagnosed individuals to model a SARS epidemic with a net inflow of individuals into a region. Ding et al. [26] came up with a simplified SEIJR version (SIJR) to analyze parameters such as transmission rate, basic reproductive number, etc., from SARS outbreaks in Hong Kong, Singapore, and Canada.

While small in volume, research on the MERS epidemic dynamics brings in some fresh perspectives in addition to the abovementioned compartmental approaches to the SARS outbreak. For example, Nah et al. [27] analyzed MERS importation through airline networks from Saudi Arabia to other countries to parameterize a hazard-based risk prediction model. Their analysis on migration patterns’ impact on infection spread is particularly interesting, as the world in 2013 was a lot more mobile than that in 2003 (the year of SARS). Their work incorporated a spatial dimension to simulate a more realistic transmission environment.

### 2.3. Review of COVID Modeling

Noticeably, considering the abovementioned coronavirus literature, these studies all add a worldwide mobile dimension to epidemic modeling, reflecting the higher level of globalization in 2020 than 2003 (the year of SARS) and even 2013 (the year of MERS). Wu et al. [3] used the SEIR model to infer the basic reproductive number and simulate the Wuhan epidemic from 31 December 2019 to 28 January 2020. Their model adds a mobile dimension of domestic and international air travel to/from Wuhan from/to other cities to forecast the national and global spread of the virus, accounting for the effect of the metropolis-wide quarantine of Wuhan and surrounding cities (starting on 23–24 January 2020). Similarly, Kucharski et al. [5] employed a stochastic dual-SEIR approach for both the Wuhan population and international travelers from Wuhan to estimate how transmission varied over time between January and February 2020 from Wuhan to international destinations, adding a mobile dimension to the outbreak dynamics. Chinazzi et al. [6] simulated the international spread of COVID-19 after the start of the travel ban from Wuhan on 23 January 2020 until 3 March 2020. They applied a SEIR variety (SLIR) to project the impact of transmissions. Their approach also introduced a mobile dimension to epidemic modeling.

### 2.4. Gaps in the Literature

From the literature review, we observe that compartmental models (and, particularly, the SEIR family) dominate epidemic modeling, and virtually all of the studies that apply compartmental models seek to estimate the transmission parameter based on extensive epidemic datasets, most of which are privately held and not freely accessible by the general public. In addition, infectious diseases spread in three dimensions: temporal, spatial, and mobile. Although epidemic datasets are time series in nature and, therefore, compartmental models already include the temporal dimension by default, they do not explicitly model the spatial and mobile dimensions without significant structural upgrade. As a consequence, these compartmental models are limited if the true transmission parameters depend upon the geographic distribution and mobility of the population, which is the case for COVID-19. Thus, the aim of our research is to develop a unified temporal–spatial–mobile parameterization process that enhances the utility of current compartmental models and, in the meantime, makes use of datasets that can be obtained in a cost-effective way.

## 3. Materials and Methods

### 3.1. Compartmental Models

The mainstream compartmental models require detailed statistics on the characteristics of an infectious disease to estimate the stochastic transmission parameters between compartments. Essentially, these models correlate tempo-spatial factors (e.g., geographic distances and contact durations) among heterogeneous subpopulations with gradient probability decays. Carefully designed transmission parameterization processes utilize Bayesian inference methods, such as Markov Chain Monte Carlo (MCMC) or Gillespie algorithm [28] simulations, to form probability density functions (PDFs) from cross-sections to estimate transmission parameters for each timestep in a compartmental time series. These need further calibration with historical transmission data to achieve a reasonable level of accuracy. However, detailed statistics on transmission characteristics are expensive, both economically and resource-wise, to collect. As an alternative, some researchers (e.g., [13]) simply assume the values of these transmission parameters to achieve cost-effectiveness.

We are particularly interested in compartmental models that cover multiple interconnected and heterogeneous subpopulations [9,25,29]. We first develop a multistep, multivariate deep learning methodology to estimate the transmission parameters and then feed them to a class of customized compartmental models to predict and simulate the development of the Omicron epidemic in China (including and excluding Hong Kong and Taiwan), Hong Kong, and Taiwan.

We establish a SIR-derived discrete time series on a daily interval as the theoretical foundation for a deep learning-enhanced compartmental model—SIRD (Susceptible–Infectious–Recovered–Deceased). A precursor to this study has been developed to predict and simulate the dynamics and development of the original COVID-19 variant in the US [30].

The SIRD construct groups a population into four compartments:

1. Susceptible (S): The susceptible population that progresses into the infectious compartment;
2. Infectious (I): The infectious individuals who are symptomatic come from the Susceptible compartment and progress into the Recovered compartment;
3. Recovered (R): The recovered individuals come from the infectious compartment and acquire lasting immunity (there has yet to be any contradiction against this assumption for Omicron);
4. Deceased (D): The deceased cases come from the infectious compartment.

The SIRD model has a discrete daily (∆t=1) multivariate time series construct given by the following matrix form:

(1) $$\left[\begin{array}{c} \begin{array}{c} S_{t+1}\\ I_{t+1}\\ R_{t+1} \end{array}\\ D_{t+1} \end{array}\right]=\left[\begin{array}{c} \begin{array}{c} 1\\ 0\\ 0 \end{array}\\ 0 \end{array}\begin{array}{c} \begin{array}{c} -\beta \\ 1+\beta -\gamma^{R}-\gamma^{D}\\ \gamma^{R} \end{array}\\ \gamma^{D} \end{array}\begin{array}{c} \begin{array}{c} 0\\ 0\\ 1 \end{array}\\ 0 \end{array}\begin{array}{c} \begin{array}{c} 0\\ 0\\ 0 \end{array}\\ 1 \end{array}\right]\left[\begin{array}{c} \begin{array}{c} S_{t}\\ I_{t}\\ R_{t} \end{array}\\ D_{t} \end{array}\right]$$

or

(2) $$\vec{V_{t+1}}=\overset{⃡}{A_{t}}\vec{V_{t}}$$

where

$$\vec{V_{t+1}}=\left[\begin{array}{c} \begin{array}{c} S_{t+1}\\ I_{t+1}\\ R_{t+1} \end{array}\\ D_{t+1} \end{array}\right],\overset{⃡}{A_{t}}=\left[\begin{array}{c} \begin{array}{c} 1\\ 0\\ 0 \end{array}\\ 0 \end{array}\begin{array}{c} \begin{array}{c} -\beta \\ 1+\beta -\gamma^{R}-\gamma^{D}\\ \gamma^{R} \end{array}\\ \gamma^{D} \end{array}\begin{array}{c} \begin{array}{c} 0\\ 0\\ 1 \end{array}\\ 0 \end{array}\begin{array}{c} \begin{array}{c} 0\\ 0\\ 0 \end{array}\\ 1 \end{array}\right]\;and\;\vec{V_{t}}=\left[\begin{array}{c} \begin{array}{c} S_{t}\\ I_{t}\\ R_{t} \end{array}\\ D_{t} \end{array}\right]$$

The Greek letters $\beta ,\gamma^{R},\;\gamma^{D}$ in Equations (1) and (2) are the “susceptible-to-infectious”, “infectious-to-recovered” and “infectious-to-deceased” transmission parameters.

Since we need to estimate the transmission parameters, we rewrite and rearrange Equations (1) and (2) in the following matrix representation:

(3) $$\left[\begin{array}{c} {∆S}_{t+1}\\ ∆I_{t+1}\\ ∆R_{t+1}\\ {∆D}_{t+1} \end{array}\right]=\left[\begin{array}{c} -I_{t}\\ I_{t}\\ 0\\ 0 \end{array}\begin{array}{c} 0\\ -I_{t}\\ I_{t}\\ 0 \end{array}\begin{array}{c} 0\\ -I_{t}\\ 0\\ I_{t} \end{array}\right]\left[\begin{array}{c} \beta \\ \gamma^{R}\\ \gamma^{D} \end{array}\right]$$

or

(4) $$\vec{{∆V}_{t+1}}=\overset{⃡}{B_{t}}\vec{{Ι}_{t}}$$

where

$$\vec{{∆V}_{t+1}}=\left[\begin{array}{c} {∆S}_{t+1}\\ ∆I_{t+1}\\ ∆R_{t+1}\\ {∆D}_{t+1} \end{array}\right],\overset{⃡}{B_{t}}=\left[\begin{array}{c} -I_{t}\\ I_{t}\\ 0\\ 0 \end{array}\begin{array}{c} 0\\ -I_{t}\\ I_{t}\\ 0 \end{array}\begin{array}{c} 0\\ -I_{t}\\ 0\\ I_{t} \end{array}\right]\;and\;\vec{{Ι}_{t}}=\left[\begin{array}{c} \beta \\ \gamma^{R}\\ \gamma^{D} \end{array}\right]$$

### 3.2. Parameterization with Deep Learning and SIRD Simulation

The transmission parameters ($\beta ,\gamma^{R},\gamma^{D}$) in Equations (1) to (4) are non-stochastic values in the temporal dimension (*t*) and stochastic variables along three “spatial dimensions,” namely, population distribution (*S*), population mobility (*L*), and population heterogeneity (*C*). A parameterization to estimate the transmission parameters at each timestep (cross-section in the multivariate SIRD construct) is, therefore, required and has the following expression:

(5) $$F_{tp}=F_{tp}\left(t,S,L,C\right)\in \left(\beta ,\gamma^{R},\gamma^{D}\right)$$

Equation (5) shows that each transmission parameter ($F_{tp}$) can be modeled in a 4-dimensional tempo-spatial framework. The parameterization process is thus to estimate the in-sample values of cross-sectional $F_{tp}$ at each timestep *t* in the SIRD time series construct and predict its out-of-sample values.

We aim to build a multistep, multivariate deep learning method to estimate the transmission parameters, utilizing both a standard deep neural network (DNN) and the advanced recurrent neural network–long short-term memory neural network (RNN-LSTM, or simply LSTM) methodologies. We propose the following steps to achieve this goal:

1. Constructing the in-sample SIRD time series using observed Omicron data;
2. Calculating in-sample daily transmission parameters from the in-sample SIRD time series constructed in Step 1;
3. Decomposing Equation (5) as
  (6) $$F_{tp}=F_{tp}\left(t,S,L,C\right)=F_{tp}\left(t\right)\Psi \left(S,L,C\right).$$
  that is, along the temporal dimension, at the given timestep *t*, the non-stochastic value of the transmission parameter is $F_{tp}\left(t\right)$; along the spatial dimensions (*S*, *L*, *C*), the cross-sectional probability distribution of the transmission parameter is $\Psi \left(S,L,C\right)$;
4. Deep learning algorithms (DNN and LSTM) are applied to fit the in-sample decomposed transmission parameters in Step 3. Deep learning is performed on both $F_{tp}\left(t\right)$ and $\Psi \left(S,L,C\right)$ to calibrate the in-sample values of $F_{tp}\left(t,S,L,C\right)$ along both temporal and spatial dimensions, respectively;
5. With the in-sample transmission parameters obtained in Step 4, the DNN and RNN-LSTM algorithms are applied again, in both progressive and recursive manners, to predict the out-of-sample transmission parameters for multiple scenarios;
6. Simulating out-of-sample Omicron dynamics recursively through the SIRD time series, using the out-of-sample transmission parameters predicted in Step 5.

The methodological innovation of our research is mainly reflected in the deep learning of the cross-sectional probability distribution $\Psi \left(S,L,C\right)$.

In general, from Equation (6), the traditional stochastic parameterization processes utilize Bayesian inference methods [31] to form PDFs on cross-sections to estimate transmission parameters for each timestep of a compartmental time series as follows:

(7a) $$F_{tp}=F_{tp}\left(t,S,L,C\right)=F_{tp}\left(t\right)\;\prod_{S}\;\left(P\left(S\right)\right)\prod_{L}\left(P\left(L\right)\right)\prod_{C}\left(P\left(C\right)\right)=\;F_{tp}\left(t\right)\Psi \left(S,L,C\right)$$

(7b) $$\Psi \left(S,L,C\right)=\prod_{S}\left(P\left(S\right)\right)\prod_{L}\left(P\left(L\right)\right)\prod_{C}\left(P\left(C\right)\right)$$

(7c) $$P\left(S\right)=p\left(S\right)e^{-\frac{1}{S}};P\left(L\right)=p\left(L\right)e^{-\frac{1}{L}};P\left(C\right)=p\left(C\right)e^{-\frac{1}{C}}$$

(7d) $$p\left(S\right)=\frac{1}{1+e^{\alpha_{S}+\beta_{S}S}};p\left(L\right)=\frac{1}{1+e^{\alpha_{L}+\beta_{L}L}};p\left(C\right)=\frac{1}{1+e^{\alpha_{C}+\beta_{C}C}}$$

In Equation (7a–d), α and β are the random variables of Geometric Brownian Motion (see, for example, [32]). S,L,C represent the “distance” between individuals in the three spatial dimensions. $p\left(S\right)$, $p\left(L\right)$, $p\left(C\right)$ are PDFs, while $e^{-\frac{1}{S}}$, $e^{-\frac{1}{L}}$, and $e^{-\frac{1}{C}}$ are discrete decay distributions. $P\left(S\right),P\left(L\right),P\left(C\right)$ are the corresponding probability distribution functions. With the actual observed values (in-sample) of S,L,C, the in-sample $p\left(S\right)$, $p\left(L\right)$, $p\left(C\right)$ that match the observed values are used as the baseline to determine the ranges of α and β; then, a Monte Carlo simulation is conducted to estimate the values of out-of-sample $p\left(S\right)$, $p\left(L\right)$, $p\left(C\right)$.

The above process of generating $\Psi \left(S,L,C\right)$, the joint probability distribution of the spatial-dimensional parameters, is the stochastic parameterization process, which requires calibration with historical transmission data to achieve a reasonable level of accuracy. However, detailed statistics on transmission characteristics are expensive, economically and resource-wise, to collect. This makes our deep learning-based parameterization approach, which uncovers hidden interconnections among observed data yet greatly reduces the dependency on data particularity, an attractive alternative.

### 3.3. Data

We collect the COVID-19 datasets from the following two sources:

1. Dataset 1: A JHU CSSE dataset (available as the Supplementary File S1. Dataset_1.csv), which tracks confirmed cases and deceased cases. We use the confirmed/deceased dataset to form training data for deep learning;
2. Dataset 2: A Tencent dataset (available as the Supplementary File S2. Dataset_2.csv), which updates daily records (confirmed, active, deceased, recovered, etc.). We use these detailed case data to construct the compartmental model.

In general, both datasets have some reporting discrepancies, with certain extreme outliers in both directions; thus, we run a 7-day moving average on the datasets to smooth out these data irregularities.

### 3.4. Modeling Methodology

**Figure 1:**
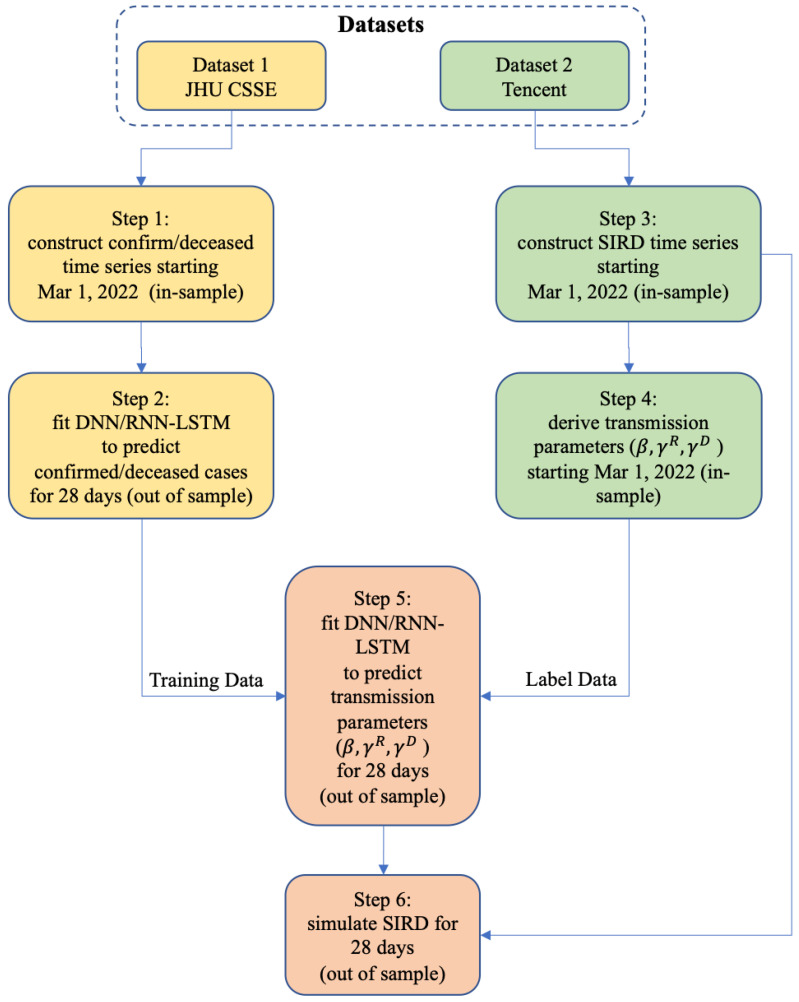
The methodology flowchart.

We then conduct the following step-by-step operations to model the Omicron epidemic in mainland China, Hong Kong, and Taiwan (Steps 1 and 2) and the whole country (all steps). Figure 1 is the flowchart to illustrate the modeling methodology.

1. We construct a confirmed/deceased time series starting from 1 March 2022 (in-sample) from Dataset 1. The date of 1 March 2022 is generally accepted as the outbreak point of the Omicron epidemic in China [13];
2. We apply two deep learning models (DNN and LSTM) to fit the confirmed/deceased in-sample time series from Step 1 and predict the further development of confirmed/deceased cases for 28 days (out-of-sample);
3. We construct an in-sample SIRD time series starting on 1 March 2022 from Dataset 2;
4. We use the in-sample SIRD time series constructed in Step 3 to come up with in-sample sequences for the SIRD daily transmission parameters (β, $\gamma^{R}$, and $\gamma^{D}$);
5. We then use the confirmed/deceased time series (in-sample and out-of-sample) from Step 2 as training data and the in-sample β, $\gamma^{R}$, and $\gamma^{D}$ sequences from Step 4 as training labels, and apply the DNN and RNN-LSTM techniques to predict β, $\gamma^{R}$, and $\gamma^{D}$ for 28 days (out-of-sample);
6. Finally, we use the predicted (out-of-sample) transmission parameters (β, $\gamma^{R}$, and $\gamma^{D}$) from Step 5 to simulate the 28-day progression (out-of-sample) of the SIRD model in a recursive manner, starting from the last timestep from the in-sample SIRD time series from Step 4;
7. We then repeat Steps 1–6 for Hong Kong and Taiwan to test the robustness of the model with data from different phases (and populations) of the epidemic.

We use MSE and RMSE as convergence criteria for the DNN and RNN-LSTM learning. We adjust the number of learning iterations (epoch) and make subjective judgements on the optimized error decay rate so that the epoch (e.g., 64, 128, etc.) is minimized to avoid computational complexity.

Several groups of researchers [3,5,6] estimate that the generation time of COVID in China is about 1 to 2 weeks, and Deng [31] forecasts 35 days and 42 days in advance for the Omicron development in the US. We find that the computational complexity for forecasting 35/42 days in advance is prohibitive, while forecasting for fewer than 14 days might not be of practical value. Therefore, we choose 28 days as a reasonable forecasting period.

## 4. Results

**Figure 2:**
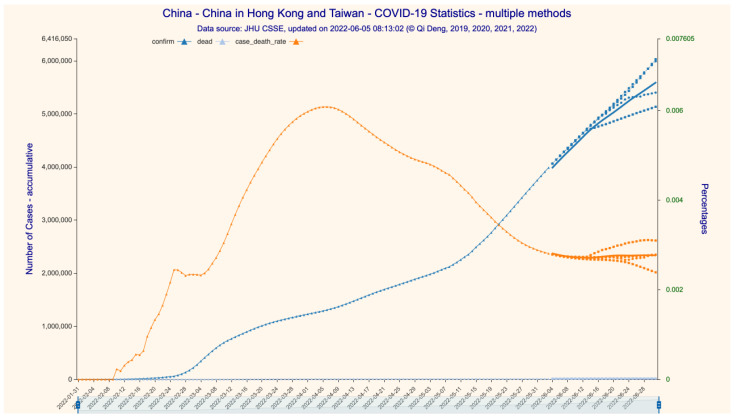
The 28-day forecast for the confirmed/deceased cases in China (incl. Hong Kong and Taiwan). Figure Legend: (1) confirm—accumulative number of confirmed infections, (2) dead—accumulative number of deaths, (3) case death rate = dead/confirm, representing overall death rate. Data source: Supplementary File S1. Dataset_1.csv.

**Figure 3:**
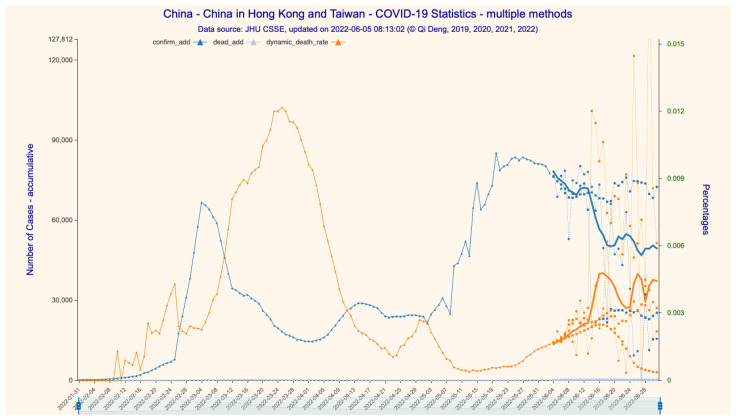
The 28-day forecast for the increase in confirmed/deceased cases in China (incl. Hong Kong and Taiwan). Figure Legend: (1) confirm_add—daily increase in the number of confirmed infections, (2) dead_add—daily increase in the number of deaths, (3) dynamic_death_rate = dead_add/confirm_add, representing the trend in death rate. Data source: Supplementary File S1. Dataset_1.csv.

**Figure 4:**
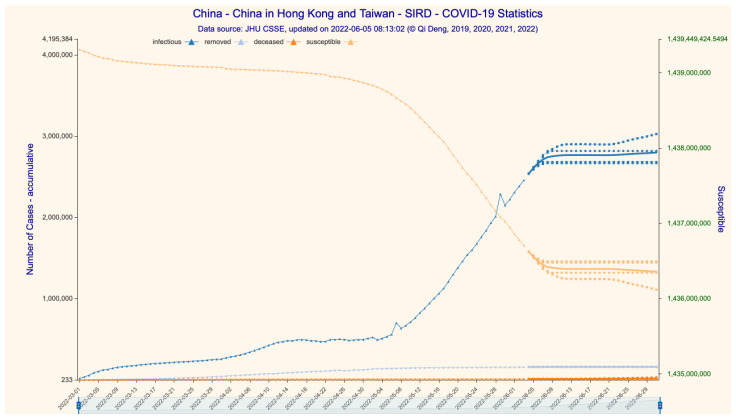
The 28-day SIRD simulation for cases in China (incl. Hong Kong and Taiwan). Data source: Supplementary File S1. Dataset_1.csv; Supplementary File S2. Dataset_2.csv.

The average results of eight models (scenarios with different learning hyperparameters) based on data up to 3 June 2022 are illustrated in Figure 2, Figure 3 and Figure 4 (28-day forecast). The predicted numbers of infections and deaths and the predicted case fatality rate (CFR) are listed in Table 1.

We predict that, over the 28-day forecast period in mainland China (excluding Hong Kong and Taiwan), the daily Omicron infection increase is between 60 and 260. On 1 July 2022, there would be 768,622 cumulative confirmed cases and 591 cumulative deceased cases, with a CFR of 0.0769%. On 1 July 2022, in Hong Kong, there would be 1,220,352 cumulative confirmed cases and 9282 cumulative deceased cases, with a CFR of 0.7606%. We further predict that the rate of daily infection increase in Hong Kong would be flat and at a very low level until at least the end of June 2022, at a value between 350 and 1100. On 1 July 2022, in Taiwan, there would be 3,842,576 cumulative confirmed cases and 4482 cumulative deceased cases, with a CFR of 0.1166%. The Omicron epidemic in Taiwan is far from being over: the rate of daily infection increase peaks by the end of May at approximately 83,000 and drops to approximately 41,750 by 1 July 2022.

Since dataset 2 only provides detailed time series case data for mainland China, we are only able to construct the SIRD time series for the whole country, not for the subregions. We predict that the numbers of cumulative confirmed/deceased cases would be 5,587,799 and 15,380, respectively, with a CFR stabilizing at 0.2752% on 1 July 2022 (Figure 2 and Figure 3). We then forecast the transmission parameters and simulate the dynamics and development of the Omicron epidemic with the SIRD time series construct (Figure 4). For the 28-day time period ending on 1 July 2022, we find that the daily infection increase has already peaked at the end of May and drops steadily to near zero around 14 June 2022, but then rises at a low level between 150 and 4000 afterwards.

We then compare our predictions against the reported (actual) data for the same 28-day time span (Table 1). The prediction errors in our model are at different levels of accuracy, depending upon regions and case types. For the number of infections, the prediction error for mainland China is the lowest at 0.04% (99.96% accuracy), which can be explained by China’s extremely strict zero-COVID-19 policy, reducing the volatility of transmission dynamics to an extremely low level. The prediction error for Taiwan is the highest at 4.51% (95.49% accuracy), as Taiwan did not take any extra measures against COVID-19 that would have interrupted the daily routines and movement of the populace; thus, COVID-19 transmission essentially followed its natural path. The prediction error for Hong Kong is in the middle at 0.62% (99.38% accuracy) because Hong Kong executed a somewhat middle-of-the-road strategy that balanced disease control and economic development. That the CFR for Hong Kong is much higher than that for Taiwan and mainland China (0.7480% vs. 0.1525% and 0.0808%, respectively) can be explained by the very low vaccination rate in Hong Kong at the time, especially among the elderly. As such, the model’s lower limit of accuracy on infection prediction is at a very respectable level of higher than 95%, and the average level of accuracy is approximately 98%.

For the numbers of deaths, the prediction errors are 5.08%, 1.03%, 24.83%, and 0.14% for mainland China, Hong Kong, Taiwan, and the whole country, respectively. The results suggest that the level of the accuracy of death prediction is generally higher with an increased population base. That the prediction error for Taiwan is the highest (24.83%) is due to the overwhelming pressure on the island’s healthcare system. The model’s lower limit of accuracy on death prediction is higher than 75%, and the average level of accuracy is approximately 92%.

## 5. Discussion and Conclusions

The mainstream compartmental models require stochastic parameterization to estimate the transmission parameters between compartments, whose determination depends upon detailed statistics on epidemiological transmission characteristics, which are expensive, economically and resource-wise, to collect. As an alternative, deep learning techniques are effective in estimating these stochastic parameters with greatly reduced dependency on data particularity.

We apply deep learning techniques as a lower-data-dependency alternative to estimate the transmission parameters of a customized compartmental model for the purposes of simulating the dynamics of the Omicron phase of the COVID-19 epidemic and projecting its further development in China. Particularly, we apply DNN and LSTM techniques to estimate the stochastic transmission parameters for a SIRD model with a discrete time series construct. We then apply DNN and LSTM deep learning techniques to fit the confirmed/deceased time series to predict the further development of confirmed/deceased cases, as well as to predict the transmission parameters (β, $\gamma^{R}$, $\gamma^{D}$) for 28 days. Finally, we use the predicted transmission parameters to simulate the Omicron dynamics for 28 days. The average levels of prediction accuracy of the model are 98% and 92% for the numbers of infections and deaths, respectively.

The effectiveness of prevalent compartmental modes depends upon the availability of detailed statistics on epidemiological transmission characteristics. As an alternative, with the introduction of the deep learning-enhanced compartmental model, we provide an effective and easy-to-implement alternative to prevailing stochastic parameterization. The deep learning techniques uncover hidden interconnections among observed data, which greatly reduces the dependency on data particularity. Our model demonstrates the efficacy and potential of applying deep learning methods in predicting the dynamics of infectious diseases beyond the current dataset (and, for that matter, COVID-19). This argument is partially supported by the fact that a precursor to this study has been developed to predict and simulate the dynamics and development of the original COVID-19 variant in the US with success [17].

## Figures and Tables

**Table 1:** Prediction accuracy.

	Predicted			Reported			Prediction Error (Absolute)	
	Infection	Death	Case Fatality Rate	Infection	Death	Case Fatality Rate	Infection	Death
China (mainland)	768,622	591	0.0769%	768,935	621	0.0808%	0.04%	5.08%
Hong Kong	1,220,352	9282	0.7606%	1,228,002	9186	0.7480%	0.63%	1.03%
Taiwan	3,842,576	4482	0.1166%	3,669,157	5595	0.1525%	4.51%	24.83%
China	5,587,799	15,380	0.2752%	5,666,094	15,402	0.2718%	1.40%	0.14%

## Data Availability

Datasets are available as Supplementary Materials.

## Supplementary Materials

The following supporting information can be downloaded at: https://www.mdpi.com/article/10.3390/bioengineering11090906/s1, File S1. Dataset_1; File S2. Dataset_2.
